# Supplementary material for: Content-rich biological network constructed by mining PubMed abstracts
Source: BMC Bioinformatics. 2004 Oct 8;5:147. doi: 10.1186/1471-2105-5-147 (PMC528731; doi:10.1186/1471-2105-5-147)
Supplement: Additional File 5 — The original Chilibot query results of the term "long-term potentiation (LTP)" and 22 other terms, limiting the latest references analyzed to the years 1990, 1995, 2000, and 2004. [file 1471-2105-5-147-S5.bz2 › chilibotAdditionalFile5/ltp2004/html/ACTININ.html]

 


**ACTININ** (Input: ACTININ ) 

---


**Google Searches:** Entire Web  | EDU domain only  | PDF files only

.

**External Links:** OMIM |
LocusLink
| Swissprot
| GeneCards

  
**Maps of ACTININ**

Simple
Complete
graph in
radiant
tree
square
layout.

**New Hypothesis !**

**Synonyms** 

- actinin   [PubMed]

**Synopsis**

- These results suggest that PI 3 kinase  [PI-3K]  binds to alpha **actinin** and regulates cytoskeletal reorganization.  Biochem J, 1994 
  [23]

- Our results suggest that  calcium influx through L type VSCCs activates two distinct signaling branches, probably in the different domains of the growth cone,i.e., calcium dependent phosphorylation of GAP 43 and MARCKS protein, and calcium dependent degradation of brain spectrin and the release of alpha **actinin** by calpain.  J Neurosci Res, 1998 
  [20]

- We conclude that the NR1 C0 region is a key site for recruiting CaMKII to the postsynaptic site, where it may act in concert with calmodulin to modulate the stimulatory role of alpha **actinin** interaction with the NMDA receptor.  J Biol Chem, 2002 
  [16]

- We suggest that the signaling pathway from the IGF IR kinase through the PI 3 kinase  [PI-3K]  to alpha **actinin** participates in the rapid organization of actin into microspikes at the cell cell junctions and leads to active cell separation.  J Cell Sci, 2002 
  [16]

- We suggest how recently discovered binding interactions could provide a structural readout mechanism the autophosphorylated state of CaMKII binds tightly to the NMDAR and forms, through CaMKII **actinin** actin 4.1 SAP97 linkages, additional sites for anchoring AMPARs at synapses.  Neuron, 2001 
  [16]

- After double immunostaining of transverse cross sections of the papillary muscles with AKAP100 plus alpha **actinin** specific antibodies or AKAP100 plus ryanodine receptor specific antibodies, confocal images showed AKAP100 localization at the region of the transverse tubule junctional sarcoplasmic reticulum.  J Cell Biol, 1998 
  [16]

- Staining of PKC and vimentin, alpha **actinin**, and vinculin suggests that PKC participates actively in the transduction of mechanical signals to the cell through focal adhesions and the cytoskeleton, although only PKC seemed to change with short time periods of strain.  J Bone Miner Res, 1994 
  [16]

- Phosphorylation of focal adhesin kinase FAK at Tyr397 and the formation of complexes between FAK and PI 3 kinase  [PI-3K]  and between alpha **actinin** and vinculin were found to correlate with type 1 pilus mediated bacterial invasion.  EMBO J, 2000 
  [15]

- Unlike the reported interaction between **actinin** and the NMDA receptor, calmodulin was found to have no effect on **actinin** binding to Kv1.5.  FEBS Lett, 2001 
  [15]

- These results indicate that residues 168 184 comprise a binding site for PIP2 in alpha **actinin**.  J Biol Chem, 1996 
  [14]

- These results provide strong evidence for the interaction between MEKK1 and alpha **actinin**.  Cell Motil Cytoskeleton, 1999 
  [14]

- Treatment of EBs from day 3 to day 7 with 50 microM LY294002 resulted in a massive loss of alpha **actinin** stained cardiomyocytes after plating the EBs for additional 7 days.  Exp Cell Res, 1999 
  [11]

- The CH domain of calponin could compete with intact calponin or alpha **actinin** for ERK binding.  Biochem J, 1999 
  [10]

- alphaactinin 2 in rat striatum localization and interaction with NMDA glutamate receptor subunits.  Brain Res Mol Brain Res, 2000 
  [10]

- Drebrin A and alpha **actinin** 2 are two major actin binding proteins in dendritic spines.  Neurosci Res, 2001 
  [10]
